# Complete Placenta Prævia

**Published:** 1882-08

**Authors:** P. W. Van Peyma


### Clinical Reports.

### Complete Placenta Prævia.

By P. W. Van Peyma, M. D.

On the evening of the 16th of May, I was called by Mrs. O., a midwife, to see Mrs. G., in labor with her seventh child. I was informed by the midwife of the abnormal condition, and upon examination with the hand entirely within the vagina, I was unable to feel any part of the child. The os uteri was dilated to a diameter of about two inches and was further dilatable.

The entire opening was covered by the placenta. The patient's condition was on the whole favorable; the pulse, though rapid and weak, was not remarkably so. The loss of blood had not been excessive. On introducing the hand, for the purpose of turning, I failed to find any part of the os uncovered, and therefore, in order to reach the cavity of the uterus, I decided to perforate the placenta with my hand. This was accomplished without great difficulty, and although it proved a head presentation, the feet were easily reached and version readily accomplished. The child and placenta were delivered at the same time. The child, although showing some signs of life, we were unable to resuscitate, its death being apparently due to loss of blood. The mother was convalescent within the usual time, and has since been perfectly well. In relation to this case I will simply add that while cases of complete placenta prævia are rare, it is my experience that partial placenta prævia is comparatively common.

